# Personalized Cancer Risk Assessments for Space Radiation Exposures

**DOI:** 10.3389/fonc.2016.00038

**Published:** 2016-02-22

**Authors:** Paul A. Locke, Michael M. Weil

**Affiliations:** ^1^Department of Environmental Health Sciences, Johns Hopkins Bloomberg School of Public Health, Baltimore, MD, USA; ^2^Department of Environmental and Radiological Health Sciences, Colorado State University, Fort Collins, CO, USA

**Keywords:** genetic susceptibility, radiation carcinogenesis, cancer risk, space radiation, cancer

## Abstract

Individuals differ in their susceptibility to radiogenic cancers, and there is evidence that this inter-individual susceptibility extends to HZE ion-induced carcinogenesis. Three components of individual risk: sex, age at exposure, and prior tobacco use, are already incorporated into the NASA cancer risk model used to determine safe days in space for US astronauts. Here, we examine other risk factors that could potentially be included in risk calculations. These include personal and family medical history, the presence of pre-malignant cells that could undergo malignant transformation as a consequence of radiation exposure, the results from phenotypic assays of radiosensitivity, heritable genetic polymorphisms associated with radiosensitivity, and postflight monitoring. Inclusion of these additional risk or risk reduction factors has the potential to personalize risk estimates for individual astronauts and could influence the determination of safe days in space. We consider how this type of assessment could be used and explore how the provisions of the federal Genetic Information Non-discrimination Act could impact the collection, dissemination and use of this information by NASA.

## Introduction

In spaceflight, astronauts are exposed to a radiation environment consisting of a uniform flux of background galactic cosmic radiation with intermittent pulses of high energy protons from solar particle events. As employers, NASA must comply with the federal Occupational Safety and Health Act, which (among other things) requires NASA to set radiation exposure limits to protect the health of astronauts on space missions (1). At the time of this writing, NASA’s approach to setting permissible radiation exposure limits is unique among federal agencies. The risk of developing a fatal cancer from radiation exposure is calculated using a regularly updated model, currently NSCR 2012 (2) as recently revised (3). Career exposures are limited to doses that will not result in more than a 3% probability of fatal cancer (risk of exposure-induced death or REID) at the 95% upper confidence interval of the risk calculation. For an individual astronaut, the risk calculation takes into account the astronaut’s age at exposure and sex, and assumes that he or she is a non-smoker. Because the risk for most radiation-induced cancers decreases with older ages at exposure and risks are greater in females than males, the effect is to allow less cumulative flight time for female and younger astronauts. Several reference missions, including a near Earth asteroid mission and Mars missions exceed the 3% REID for fatal cancer; so do multiple ISS missions exceeding a total duration of about 24 months for male astronauts and about 18 months for female astronauts during solar minimum (3, 4). This article examines whether possible personalized risk approaches might be used to characterize these risks. It is important to point out that these excess risks also raise important ethical issues that are beyond the scope of this article (5).

The inclusions of age at exposure, sex, and smoking status in setting radiation dose limits can be viewed as steps toward personalizing risk assessments. There are additional approaches, either feasible or currently available, that could further personalize these assessments. Personalized risk calculations could potentially be used for pre-employment screening to select crew members for particular flights or could be provided to crew members and their flight surgeons for personal medical counseling with confidentiality safeguards. The legal issues raised by each of these uses are discussed below.

This article considers evidence that inter-individual differences contribute to cancer risk from radiation exposures, how these differences can be detected and how the information might be used. In addition, this article discusses how potential approaches for the detection of inter-individual differences in susceptibility to radiogenic cancer could impinge on the federal Genetic Information Non-discrimination Act of 2008 (GINA) and explores whether an employer (i.e., NASA) could lawfully use the information in employment or work assignment decisions.

Cancer risk is not generic. There are specific cancers that pose the greatest risks of exposure induced death. Using calculated REIDs from NSCR 2012 for 45-year-old male and female astronauts [Tables A3 and A7 in Ref. (2)], the greatest risks are for lung, stomach, colon, ovarian, breast, liver, and bladder cancers for females, and lung, colon stomach, bladder, liver, and prostate cancers for males. Leukemia is also a risk for both sexes. Each of these tumor types is likely unique in the extent to which susceptibility to them differs between individuals and in their amenability to preclinical detection.

## Approaches

### Personal and Family Medical History

Relative risk and absolute risk models and combinations of the two are used in risk calculations. Relative risk is calculated as a dose-dependent multiple of the background incidence of a cancer, whereas absolute risk is an added number of cases per unit dose that is independent of the background incidence. Which model best fits epidemiological data depends on the tumor type being modeled. The relative risk model assumes that radiation increases the incidence of spontaneous tumors and implicit in that assumption is that radiogenic cancers are the same or nearly the same as their spontaneous counterparts. There is evidence that the relative risk model reflects biological reality for at least some radiogenic cancers. The few radiogenic tumors that have been characterized carry the same cytogenetic and molecular aberrations as a subset of spontaneous tumors of the same histotype (6–11). For example, sporadic acute myeloid leukemias (AML) have a range of recurrent chromosomal aberrations, predominantly translocations. However, radiation-induced AML are generally associated with deletions on chromosome 5 and/or 7 (11), cytogenetic lesions that occur in only a few percent of sporadic AML (12). The most plausible explanation is that radiation can contribute a step or steps to some of the pathways leading to sporadic leukemias (e.g., those involving chromosome 5 or 7 deletions), but radiation is ineffective in complementing other leukemogenic pathways.

If the goal is to move from a population-based risk calculation to a personalized risk calculation for setting permissible space radiation doses for an individual astronaut, one possible approach would be to use the astronaut’s background risk in place of the population background risk as the baseline for relative risk calculations. Individuals differ in their susceptibility to spontaneous cancers due to a number of factors related to lifestyle, genetic background, and poorly characterized environmental exposures. For example, a family history of some cancers (e.g., colorectal and breast cancer) confers greater risk. An individual with a first degree relative diagnosed with a colorectal cancer (CRC) is 2.4-fold more likely to develop CRC than someone without an affected relative (13). For that matter, the monozygotic (identical) twin of a man with colon cancer has about a 7-fold greater risk of developing colon cancer than a man with an unaffected twin, for woman with an affected monozygotic twin the risk is about 14-fold (14).

Risk calculators are readily available for some sporadic cancers. Several have been developed that assess individual breast cancer risk based on combinations of inputs on family history of breast and ovarian cancer, current age, race or ethnicity, breast biopsy history, age at menarche, breast tissue mammographic density, and reproductive history (15). Potentially, some of these inputs might be useful in personalizing radiogenic breast cancer risk calculations. Risk calculators are also available for CRC (16–18). The information input into these calculators includes family history, sex, current age, race and ethnicity, diet, body mass index, screening history, polyp history, use of aspirin, non-steroidal anti-inflammatory drugs, oral contraceptives and estrogen replacement, physical activity, smoking, and alcohol consumption.

#### Synopsis

Individual risk of radiation carcinogenesis might be more accurately calculated by including family history of cancer and/or personal medical history including a history of colon polyps or breast biopsies. An assumption in this approach is that breast or colon cancer risk from space radiation exposure can be predicted, at least partly, from background risk by a transfer model incorporating multiplicative risk. In the current risk model, this assumption is made for CRC through the use of a mixture model (0.7 multiplicative and 0.3 additive) [Ref. (2), p. 80]. An additive model is used for breast cancer because it better fits results of a meta-analysis, not because of a biological basis.

### Detection of Preneoplastic Cells and Dormant Microtumors

Preneoplastic cells and dormant microtumors are frequently detectable in clinically normal individuals (19, 20), and it has been proposed that radiation exposure can lead to their promotion and/or progression. The best evidence for this comes from studies of leukemia. In 2005, Nori Nakamura advanced the hypothesis some individuals harbor clones of hematopoietic cells with preleukemic mutations and consequently are susceptible to radiogenic leukemias (21). The putative mechanism is that radiation exposure induces additional leukemogenic mutations in the preleukemic cells leading to overt disease. Nakamura’s hypothesis is based on epidemiological investigations of leukemia in atomic bomb survivors and the findings of Mori et al. (22) showing that leukemia relevant translocations can be detected in the blood of about 1% of newborns, the vast majority of whom will never develop leukemia.

The detection of preleukemic cells in peripheral blood samples from some clinically normal individuals has been extended to adults [e.g., Ref. (23–25)]. Data from large-scale whole exome sequencing studies using peripheral blood cells as a DNA source have been mined to identify individuals that carry clonal expanded somatic mutations in leukemia related genes. The frequency of people harboring these cells increases with age and is associated with an increased risk of hematopoietic cancer (26, 27). That increased risk suggests that at least some of the mutations detected in mature circulating blood cells occurred in stem or progenitor cells primitive enough to undergo leukemic transformation.

Perhaps the best evidence for the existence of preleukemic cells that can be driven to complete leukemic transformation by exposure to a genotoxic agent comes from recent observations by Wong and coworkers (28). Radiation-induced AML commonly carry *TP53* mutations. Wong found that two patients who developed AML following cytotoxic chemotherapy had identical *TP53* mutations in their leukemic cells and in blood samples collected prior to therapy. The likely explanation is that preleukemic cells (those with *TP53* mutations) progressed to frank leukemia as a consequence of cytotoxic chemotherapy, and the inference is that radiation exposure could have a similar effect.

Screening individuals for preleukemic cells using peripheral blood samples can be accomplished with existing technologies, either SNP arrays for genomic gains or losses or uniparental disomy, or next generation sequencing for defined mutations in clonal populations.

Some pre-invasive tumors can be detected *in situ*, with mammography for the detection of ductal carcinoma *in situ* and colonoscopy for the detection of adenomatous polyps being commonly used screens. Whether these neoplasias can be driven to malignancy by radiation exposure are unknown at this time and, consequently, the value of pre-exposure screening to lower radiogenic cancer risk is also unknown.

New early detection methods for a range of cancers are being clinically evaluated, and some examples that are relevant for tumor types of greatest interest for space flight are briefly mentioned here. Promising results have been reported for a CRC early detection test based on the identification of mutant *KRAS* sequences in DNA from tumor cells shed into stool. The assay is less intrusive than colonoscopy and therefore more likely to be used. Mammary epithelial cells are accessible for cytological screening for premalignant cells (29), and the test is offered to women at high risk for breast cancer. Its predictive value has not yet been established, but there are ongoing investigations on this approach including the incorporation of biomarker detection in the test. Low-dose computerized tomography (LDCT) lung cancer screening has been shown to decrease lung cancer mortality in heavy smokers or former smokers (30). LDCT frequently detects lesions in non-smokers, but whether these lesions are dormant microtumors that can be promoted by radiation exposure is unknown.

#### Synopsis

Assays are currently available or in development for the detection of preneoplastic cells or dormant microtumors that could potentially undergo promotion or progress to frank cancer as a consequence of radiation exposure. Individuals with these incipient malignancies may be at higher risk for radiogenic cancer than those without. The various testing procedures involve simple imaging, the detection of overexpressed or aberrant proteins, or the detection of somatic mutations in DNA from cells collected using minimally invasive techniques. The potential of premalignant cells or dormant microtumors to progress as a consequence of radiation exposure is not known.

### Phenotypic Assays of Sensitivity

The development of cell-based assays to identify radiation oncology patients sensitive to normal tissue injury has been an area of active research for many years. A logical extension of this research would be the development of assays for the identification of individuals susceptible to radiogenic cancers (or treatment-induced second malignant neoplasms in the context of radiation oncology). Cells collected from different individuals and irradiated *ex vivo* vary in their radiation responses as measured by endpoints putatively related to cancer such as clonogenic survival, DNA repair efficiency, transcriptional changes, number of cytogenetic aberrations, and proportion of cells undergoing apoptosis. Whether the inter-individual differences for any of these radiobiological endpoints predict inter-individual differences in susceptibility to radiogenic cancer is still speculative, but two assays are particularly interesting because they identify a sizable proportion of the population as being mildly radiation sensitive and are associated with sporadic cancer risk.

The G2 chromosomal radiosensitivity assay, which measures chromosome aberrations in cells irradiated in the G2 phase of the cell cycle, identifies about 5–10% of clinically normal individuals and about 40% of breast cancer patients as having enhanced chromosomal radiosensitivity (31, 32). The low dose rate (LDR) gamma-H2AX assay is based on quantifying residual DNA double strand breaks in cultured cells that have been irradiated at LDR. Fibroblasts from about 40% of clinically normal individuals fall in the mildly sensitive range, as do individuals heterozygous for *ATM* mutations, a group that has an elevated risk of breast cancer. Hereditary retinoblastoma patients who are at risk of second malignancies in the treatment field if they are treated with radiotherapy are also mildly sensitive in this assay (33, 34).

Human tumors and some human normal tissues can be propagated long term in immunosuppressed mice. Mice carrying human tumors from individual patients (patient derived xenografts) have been used to test the efficacies of alternative treatment regimens with the aim of tailoring treatment to specific tumors, thus personalizing cancer therapy. An obvious next step is the use of mice harboring normal human tissues to personalize radiogenic cancer risk assessments. The advantage of irradiating human tissue samples maintained in mice as compared to irradiating tissue samples in culture is that the tissue samples in mice would be in a more physiological setting and could be assayed long after irradiation. For example, NASA is currently supporting research that involves irradiating mice with human hematopoietic systems (so-called “humanized” mice) and monitoring the human cells for leukemia related endpoints. While this research is designed to explore the effects of simulated space radiation on the human hematopoietic system, it raises the possibility of using the same system to assess individuals for susceptibility to radiation-induced leukemia. Mice can be humanized using hematopoietic stem cells mobilized into the peripheral blood of donors by treatment with GCSF. These mice could be exposed to radiation and their human hematopoietic cells monitored for preleukemic changes such as mutations or chromosomal aberrations associated with leukemia with the goal of identifying donors whose hematopoietic cells had higher or lower frequencies of such changes.

#### Synopsis

Assays are under development that would use cells collected from individuals and irradiated *ex vivo* to determine susceptibility to radiogenic cancer. The endpoints in these assays will not be cancer *per se*, but surrogates for cancer susceptibility such as persistent DNA repair foci, chromosomal aberrations, or tumor associated mutations.

### Genotypic Assays of Sensitivity

It has been known at least since the mid-1950s that some murine inbred strains are more susceptible to specific radiogenic cancers than others (35). The most likely explanation for the strain differences in susceptibility is the genetic differences between the strains, an explanation that is strongly supported by the identification of some of the genetic polymorphisms responsible (36–38). There are multiple lines of evidence that the genetic susceptibility to radiation-induced cancers observed in mice extends to humans.

It is fairly straightforward to demonstrate that humans differ for radiation responses and the differences are, in part, heritable. Twin studies show a greater concordance for radiobiological endpoints between monozygotic twin pairs than between dizygotic twin pairs. The reasoning behind these studies is that monozygotic twin pairs share their entire genome whereas dizygotic twin pairs share only about half of their genomes, but both monozygotic and dizygotic twin pairs share the same environments. A greater concordance for a trait, such as the percentage of lymphocytes that undergo radiation-induced apoptosis, between monozygotic twin pairs than dizygotic twin pairs would be due to their greater genetic similarity. The endpoints that have been assayed and found to be under genetic control are chromatid breaks following irradiation of PHA-stimulated peripheral blood cells (39), radiation-induced apoptosis (40, 41), and radiation-induced cell cycle delay (41). These endpoints are potentially related to radiation carcinogenesis, but the findings are only suggestive that susceptibility to radiation-induced cancer is a heritable trait.

Perhaps the first epidemiological data suggesting there might be a heritable component to susceptibility to radiation-induced cancer was the finding of a high risk for breast cancer diagnosed before age 35 in A-bomb survivors suggesting interaction between radiation exposure and genetic susceptibility to early onset breast cancer (gene and radiation interaction) in a subgroup of women (42). More recently, a study of families in which multiple members had been irradiated for treatment of tinea capitis found familial aggregation of radiation-associated meningiomas (43) suggestive of genetic susceptibility.

Evidence for genetic susceptibility to radiogenic cancers also comes from clinical observations of patients with rare, heritable cancer syndromes. In these examples, increased risks of radiation-induced cancers are linked to mutations (albeit rare mutations) in known genes. Hereditary retinoblastoma patients have a high incidence of sarcomas, which is further elevated by radiotherapy (44–46), children with neurofibromatosis type I treated with radiation for optic pathway gliomas are at increased risk for second nervous system tumors (47), and Gorlin’s syndrome patients treated with radiotherapy develop basal cell carcinomas in the treatment field (48). The early onset and high penetrance of retinoblastoma makes it highly unlikely anyone with the heritable form of the disease would be selected for the astronaut corps (though *de novo* mutations resulting in somatic mosaicism mean the possibility cannot be completely excluded). While the association of some rare heritable syndromes with increased risk for radiation-induced cancers is interesting, the real questions are whether susceptibility occurs in the absence of readily identifiable syndromic disease in clinically unremarkable individuals and whether susceptible individuals are extremely rare or common.

Some common genetic polymorphisms associated with increased or decreased risks of radiogenic cancers have been identified in genetic association studies (49–54). A limitation of this approach is that the polymorphisms detected are limited to those selected for the studies, which are in genes known to be mutated in cancer or related to response to ionizing radiation-induced DNA damage.

Genome-wide association studies (GWAS) avoid the bias toward genes considered likely to influence radiosensitivity by screening the entire genome. However, GWAS studies of spontaneous cancers typically yield modest risk estimates, an observation fueling skepticism about their use in studies of radiogenic cancers. There are two reasons why GWAS associations in radiation-induced cancers may prove to be stronger. The first is that associations become stronger as the tumor subtype is more rigorously defined. There is reason to believe that radiogenic tumors only arise along a subset of oncogenic pathways (see the example of radiation-induced AML above), so GWAS associations for radiogenic tumors may be stronger because these tumors are genetically less diverse. The second reason to expect stronger associations with radiogenic than spontaneous tumors is that association studies for adverse drug reactions often yield strong associations with relatively few cases. An explanation that has been advanced for this observation is that a single strong environmental input decreases the background of other environmental causes that may operate in conjunction with other susceptibility loci (55). For example, in a study of Hodgkin’s lymphoma patients treated with radiotherapy, Best et al. identified a haplotype on chromosome 6q21 that was strongly associated with second malignant neoplasias. *PRDM1* emerged from the study as a candidate gene (56).

Whether genotypic assays of radiosensitivity can improve the precision of risk assessment will depend on a number of factors. One is the extent to which heritable sequence variants determine cancer risk from high LET exposures. High LET radiation exposures result in more complex molecular lesions that are less amenability to repair [see Ref. (2) section 5.2]. Thus, it could be argued that sequence variants that result in subtle differences in DNA repair and damage response pathways would have a lessor impact on high LET radiation carcinogenesis. However, there are profound murine and rat strain (or stock) differences in susceptibility to specific tumor types induced by high LET radiation and at least one polymorphism controlling high LET carcinogenesis has been identified (37). These observations point to a role for sequence variants in determining high LET radiation risks.

#### Synopsis

Polymorphisms in the human genome have been associated with risks for radiogenic cancers in atomic bomb survivors, radiological technologists, radiotherapy patients and people with environmental radiation exposures. Whether genotyping for these susceptibility associated polymorphisms and others that are sure to be discovered in the future will identify individuals at higher risk for cancer from the types of radiation exposures experienced space flight is currently unknown.

### PostFlight Monitoring

The NASA REID for radiation-induced cancer is not for all cancers, but rather for fatal cancers. Early detection reduces mortality for some tumor types [for the influence of tumor stage on mortality see Ref. (2), p. 51]. Regular postflight early detection cancer screening might therefore be expected to lower the risk of cancer death as a consequence of space radiation exposure assuming, of course, that radiogenic cancers are similar to their spontaneous counterparts. Early detection screens for breast, colorectal, and prostate cancer are already a routine part of medical care in the US. The relative benefits and risks of mammography screening for breast cancer, particularly before 50 years of age, and of prostate-specific antigen-based screening for prostate cancer are contentious. However, colonoscopy screening with polypectomy demonstrably reduces CRC incidence and mortality in patients with Lynch Syndrome, a heritable CRC syndrome (57–60), and also reduces sporadic CRC deaths (61). Progression from adenoma to carcinoma is accelerated in syndromic CRC, so patients with Lynch syndrome are screened at 1- or 2-year intervals. Whether standard screening intervals would be adequate to reduce CRC risk for an individual with a history of sizable exposures to space radiation is unknown.

Based on the National Lung Screening Trial (NLST), LDCT lung cancer screening decreases lung cancer mortality in current and former smokers (30). However, LDCT is not yet a routine test, it requires a high level of expertise to perform. Also, there are risks from overdiagnosis and false-positive results. These risks are important considerations because the benefits of LDCT were assessed in smokers and former smokers that varied widely in their risks for lung cancer (62) but were generally at much higher risk of lung cancer than that calculated for astronauts exposed to even maximum permissible radiation doses [Tables A1 and A5 in Ref. (2)]. An added consideration, which may be particularly relevant if LDCT were used to screen radiation exposed individuals, is additional radiation exposure from the scans themselves (63, 64).

#### Synopsis

Early detection can lower mortality for some tumor types. This reduced mortality can be incorporated in the current NASA risk model through adjustments to the incidence to mortality ratios for different tumor types. Doing so assumes that early detection of radiation-induced tumors leads to the same reduction in mortality as for sporadic tumors and that astronauts and former astronauts actually undergo early detection screenings.

## Applicability of Gina to Personalized Cancer Risk Approaches

As a federal agency, NASA is required to furnish its employees with a workplace that is free from recognized hazards such as ionizing radiation that are causing, or likely to cause, death or serious physical harm (65). NASA is also required to establish and operate an occupational safety and health program to protect workers. Recognizing the unique needs of space exploration, the federal Occupational Safety and Health Administration (OSHA) granted NASA a waiver from ground based radiation standards while requiring it to establish supplemental standards appropriate for space missions (1, 66). NASA’s Office of the Chief Medical and Health Officer is responsible for setting these standards, and issued a series of documents including NASA Procedural Requirements (NPR) 8900.1A (NASA Health and Medical Requirements for Human Space Exploration) and NPD 8900.5B (Health and Medical Policy for Human Space Exploration) in response (67, 68). In addition, NASA STD-3001, chapter 6, explicitly addresses ionizing protection in space environments (69).

### Overview of GINA

The GINA, codified as 42 US §§2000ff, is a federal law that prohibits employment discrimination on the basis of genetic information (70). This statute has two major sections. Title I covers group health plans and insurers. Title II covers employers, such as NASA, and prohibits them from discriminating against employees and job applicants based on genetic information. It also prohibits employers from collecting genetic information, except under very limited circumstances. The US Equal Opportunity Employment Commission (EEOC), an independent commission charged with enforcing federal laws against job discrimination, oversees GINA and has issued regulations to implement it (see 29 CFR §§1635.1 to 1635.12) (71). This article focuses on Title II of GINA and examines how its provisions could impact the implementation of the four approaches to personalized cancer risk assessments, summarized in Table 1 and set out earlier in this article.

**Table 1:** **Summary of approaches to personalized cancer risk assessments**.

Approach	Underlying concept	Examples of marker or data used
Personal/family medical history	For some tumor types radiogenic cancer risk is determined, in part, by background risk	Family or personal history of cancer, intestinal polyps, breast biopsies
Detection of preneoplastic cells, dormant microtumors	Preneoplastic cells and microtumors can be detected in clinically normal individuals; some of these can undergo promotion or progression due to radiation exposure	Blood or tissue aspirates assayed for cancer related mutations or biomarkers; *in situ* detection of pre-invasive tumors by imaging
Phenotypic assays of sensitivity	Radiogenic cancer susceptibility can be due to a number of genetic and non-genetic causes; *ex vivo* radiosensitivity assays of cells or tissues are agnostic in regard to the cause of susceptibility	LDR gamma-H2AX and G2 chromosomal radiosensitivity assays using peripheral blood cells or fibroblasts; cancer biomarker detection in cells or tissues irradiated in humanized mice
Genotypic assays of sensitivity	Individuals vary in their susceptibility to radiogenic cancer due to their genetic backgrounds	Genomic sequence polymorphisms

This statute and its implementing regulations are relatively new, and interpretation of its provisions is evolving [e.g., Ref. (72)]. The discussion of the applicability of GINA to NASA is based on the information available at the time of publication, and it is possible that subsequent events, especially litigation brought under GINA, could impact the way in which GINA is interpreted. The analyses are based on generalized circumstances and are not intended to provide legal advice.

One of the central provisions of GINA is that “it is an unlawful employment practice for an employer to request, require, or purchase genetic information with respect to an employee” [42 USC §2000ff-1(b)]. This provision turns on how the statute defines the term “genetic information.” Under GINA, this term means information about an individual that includes “(i) such individual’s genetic tests, (ii) the genetic tests of family members of such individual, and (iii) the manifestation of a disease or disorder in family members of such individual” [42 USC §2000ff(4)]. In addition, the term “genetic test” is “an analysis of human DNA, RNA, chromosomes, proteins, or metabolites, that detects genotypes, mutations, or chromosomal changes” [42 USC 2000ff(7)].

### Collecting and Using Personalized Information for Space Radiation Risk Assessments

This article discusses four approaches to supplement personalized cancer risk assessments. The first approach would incorporate family and personal medical history, especially for breast cancer and CRC, into these assessments. The second approach would collect information about microtumors or preneoplastic cells that could undergo promotion and/or progression by radiation exposures to malignancies. The third and fourth approaches would rely on evaluations of radiosensitivity based on genotypic and phenotypic assays. Each of these approaches would seem to trigger the collection of genetic information under GINA and therefore would most likely be prohibited under the statute. The first approach falls squarely within GINA’s prohibition of collecting personal and family medical information. The second approach would rely on the evaluation of cells and tissues based on their mutations, which also is a prohibited activity under GINA. The third and fourth approaches are keyed to radiosensitivity or genotype to phenotype associations, which again would require the collection of data that falls squarely within GINA’s purview. In summary, based on GINA’s intent and its statutory language, it appears that the data and methodologies set forth in this article that would be needed for personalization of cancer risk assessment would be prohibited by GINA. More specifically, under GINA it seems clear that NASA could not collect nor use to make employment decisions, information based on personalized cancer risk assessments that use the approaches set out in Table 1.

Two potential uses of more personalized cancer risk assessments are to screen NASA applicants and reduce employment risks to astronauts who are sent on space missions. GINA prohibits the use of genetic information and genetic tests for pre-employment screening. As this article points out, at present NASA’s approach to setting permissible exposure limits relies on a cancer risk assessment model that takes into account the astronaut’s age at exposure, sex, and smoking status. From a scientific perspective, this article suggests that among the steps to model improvement would be to utilize the increasingly powerful and more precise technologies that employ what, under GINA, would be classified as “genetic information” and “genetic tests.” Construing the statute as currently interpreted, it would seem that collecting and using this information would contravene this law and its regulations.

### PostFlight Monitoring and GINA

This article also suggests that postflight monitoring is potentially beneficial for astronauts. Such monitoring could result in reduced mortality for some tumor types, because early detection of tumors or pre-cancerous conditions could mean more effective, and timely, intervention. In this regard, GINA contains an exception to the collection of genetic information for employers who want to collect such information to assess the biological effects of toxic substances in the workplace. The employer can offer health and/or genetic services to employees in the form of a confidential wellness and/or counseling program. GINA requires that such information can be collected and used if the employee provides voluntary written authorization before collection; only the employee and family members and the genetic counselor receive this information; and that the individual genetic information not be disclosed to employers [42 USC §2000ff-1(b)(5)]. Under this section of GINA, it might be possible to collect and use the type of genetic information that is contemplated by the approaches outlined in this article. Such information might be disclosable to NASA “only in aggregate terms that do not disclose the identity of specific employees” [42 USC §2000ff – 1(b)(5)(E)].

## Author Contributions

MW provided the discussion of various approaches available to personalize risk assessment for radiation carcinogenesis. PL evaluated the impact of the Genetic Information Nondiscrimination Act on the use of these approaches.

## Conflict of Interest Statement

The authors declare that the research was conducted in the absence of any commercial or financial relationships that could be construed as a potential conflict of interest. The reviewer, Eliedonna Cacao and handling Editor, Francis A. Cucinotta declared their shared affiliation, and the handling Editor states that the process nevertheless met the standards of a fair and objective review.
